# A Lipid-Nanosphere-Small MyoD Activating RNA-Bladder Acellular Matrix Graft Scaffold [NP(saMyoD)/BAMG] Facilitates Rat Injured Bladder Muscle Repair and Regeneration [NP(saMyoD)/BAMG]

**DOI:** 10.3389/fphar.2020.00795

**Published:** 2020-06-04

**Authors:** Chongrui Jin, Nailong Cao, Jianshu Ni, Weixin Zhao, Baojun Gu, Weidong Zhu

**Affiliations:** ^1^Department of Urology, Shanghai Jiao Tong University Affiliated Sixth People’s Hospital, Shanghai, China; ^2^Department of Urology, Shanghai Eastern Urological Reconstruction and Repair Institute, Shanghai, China; ^3^Wake Forest Institute for Regenerative Medicine, Winston-Salem, NC, United States

**Keywords:** nanospheres, adipose-derived stem cells, bladder regeneration, bladder acellular matrix graft, saRNA-MyoD

## Abstract

**Background:**

Bladder tissue engineering is an excellent alternative to conventional gastrointestinal bladder enlargement in the treatment of various acquired and congenital bladder abnormalities. We constructed a nanosphere-small MyoD activating RNA-bladder acellular matrix graft scaffold NP(saMyoD)/BAMG inoculated with adipose-derived stem cells (ADSC) to explore its effect on smooth muscle regeneration and bladder repair function in a rat augmentation model.

**Methods:**

We performed many biotechniques, such as reverse transcriptase-polymerase chain reaction (RT-PCR), Western blot, MTT assay, HE staining, masson staining, and immunohistochemistry in our study. Lipid nanospheres were transfected into rat ADSCs after encapsulate saRNA-MyoD as an introduction vector. Lipid nanospheres encapsulated with saRNA-MyoD were transfected into rat ADSCs. The functional transfected rat ADSCs were called ADSC-NP(saMyoD). Then, Rat models were divided into four groups: sham group, ADSC-BAMG group, ADSC-NP(saMyoD)/BAMG group, and ADSC-NP(saMyoD)/SF(VEGF)/BAMG group. Finally, we compared the bladder function of different models by detecting the bladder histology, bladder capacity, smooth muscle function in each group.

**Results:**

RT-PCR and Western blot results showed that ADSCs transfected with NP(saMyoD) could induce high expression of α-SMA, SM22α, and Desmin. At the same time, MTT analysis showed that NP(saMyoD) did not affect the activity of ADSC cells, suggesting little toxicity. HE staining and immunohistochemistry indicated that the rat bladder repair effect (smooth muscle function, bladder capacities) was better in the ADSC-NP(saMyoD)/BAMG group, ADSC-NP(saMyoD)/SF(VEGF)/BAMG group than in the control group.

**Conclusions:**

Taken together, our results demonstrate that the NP(saMyoD)/SF(VEGF)/BAMG scaffold seeded with ADSCs could promote bladder morphological regeneration and improved bladder urinary function. This strategy of ADSC-NP(saMyoD)/SF(VEGF)/BAMG may has a potential to repair bladder defects in the future.

## Introduction

Reconstruction of the bladder is imminent in various congenital and acquired urinary tract diseases, such as neuro-bladder, bladder cancer, and congenital bladder abnormalities (Lam Van Ba et al., 2015). Bowel segment grafting has been popular for decades as the gold standard for augmentation cystoplasty or neo-bladder creation. Due to the appropriate mechanical endurance, accessibility and anatomical vascularization of the intestinal wall, it is a justified choice for bladder reconstruction. However, there are still many adverse effects, including urolithiasis, urinary tract infections, secondary malignancies, and electrolyte imbalance (Xiao et al., 2017). In the context of the imminent need for new alternatives to entero-cystoplasty, the augmentation of tissue-engineered (TE) bladder brings a new approach to bladder reconstruction (Adamowicz et al., 2019).

The basic principle mode of TE combines biologically active seed cells with scaffold materials to form a specific active material, which is acceptable for the body to promote the regeneration of bladder tissue effectively for bladder regeneration (Ardeshirylajimi et al., 2018). Among them, scaffolds and seed cells are two core elements of tissue engineering. Bladder acellular matrix graft (BAMG) has been proposed as a preferable scaffold with the same extracellular matrix composition, mechanical properties and complexity, comparing with native tissue (Coutu et al., 2014). In our previous work, we encapsulated vascular endothelial growth factor (VEGF) with silk fibroin (SF) fiber. Then we performed coaxial electrospinning technique to develop SF(VEGF)/BAMG ford a sustained-release system of VEGF, and verified its role in stimulating the growth of urethra epitheliums and smooth muscle cells (Zhu et al., 2016).

In recent years, stem cells have been widely used in tissue engineering as seed cells (Mousa et al., 2015). Adipose tissue-derived stem cells (ADSCs) are one of the most easily acquired adult stem cells and have good proliferation and differentiation ability on the surface of BAMG (Wang et al., 2019).However, some researchers showed that only a small number of ADSCs can differentiate into smooth muscle cells, and most ADSCs remain undifferentiated (Pokrywczynska et al., 2018), so increasing myogenic differentiation of ADSCs will help to reconstruct bladder function. MyoD is a member of the MRFs (myogenic regulatory factors) family of myogenic regulators. Several studies have confirmed that terminally differentiated mature adipocytes can promote myogenic proliferation through MyoD (Kocaefe et al., 2005; Kabadi et al., 2015; Wang C. et al., 2017). At present, the commonly used method is to simply adsorb the growth factor on the material. From the related literature reports and clinical application results, this method has great deficiencies: First, the combination of associated growth factors and biological materials is challengeable, owing to its diffusion degradation and unstable function in human body (Chen et al., 2014). Therefore, it is necessary to explore the application of a new method that applies MyoD in a RNA activation (RNAa) provides a new way to promote upregulation of endogenous gene expression. However, most of the nucleic acid substances are polyanions, which are difficult to penetrate the cell membrane, less intracellular accumulation, and easily degraded by nuclease during transport. Nanogene introduction vectors have attracted extensive attention due to the advantage of low toxicity, nonimmunogenicity, large loading capacity, and ease of preparation (Yang et al., 2015). Among them, lipid nanospheres (NPs) are currently the most widely used developed carrier, compared with other inorganic nanocarriers and viral-based systems, lipid nanocarriers have good biocompatibility and biodegradability (Vargas and Shon, 2019).

Based on our previous silk fibroin NPs SF(VEGF), the lipid NPs were used as the carrier of saRNA-MyoD to form a composite scaffold with BAMG, and inoculated into ADSCs. In the rat bladder defect model repair and functional recovery, trying to explore its application value for the clinical study and try to solve the current clinical bladder reconstruction problems.

## Materials and Methods

### ADSCs Isolation and Culture

Fifty- to sixty-gram Sprague Dawley (SD) rats (purchased from the Animal Center of Shanghai Jiaotong University) were anesthetized with 10% chloral hydrate, and the inguinal subcutaneous fat was excised and soaked three times with 0.25% chloramphenicol. The tissue was then minced and digested with 0.1% type I collagenase (Sigma-Aldrich Co. LLC., MO, USA) for 45 min at 37°C, filtered with a 200-mm nylon strainer (BD Falcon, Corning Inc., NY, USA) and collected by centrifugation (1,500 r/min, 10 min, 37°C). The upper layer of fat and the supernatant were removed and cultured in DMEM (Gibco, Thermo Fisher Scientific Inc.) supplemented with 10% FBS (Gibco, Thermo Fisher Scientific Inc., MA, USA) at 37°C in a 5% CO_2_. Forty-eight hours later, the cells were washed with PBS and digested with 0.25% trypsin and passaged with a ratio of 1:3 upon reaching 80%–90% confluence. The culture medium was changed every 2 days. The second passage of ADSCs was identified by flow cytometry analysis of the marker of CD34, CD90, CD29, and CD105.

### Synthesis and Selection of MyoD saRNA

DsRNAs targeting the MyoD promoter were designed and synthesized by Thermo Fisher Scientific Co., Ltd, China. Then, MyoD saRNA was transfected into ADSCs with Lipofectamine™ RNAiMAX (Thermo Fisher Scientific Co., Ltd, China) following the manufacturer’s instruction. Seventy-two hours after transfection, cells were collected to extract total protein or RNA. The effective cells were analyzed by RT-PCR and western blot and picked with statistical analysis.

The saRNA sequences are as follows: control saRNA forward: 5′-UUCUCCGAACGUGUCACGUTT-3′; control saRNA reverse: 5′-ACGUGACACGUUCGGAGAATT-3′. MyoD saRNA forward:5′-UGCCUGGUAUCCCUACAAAtt-3′; MyoD saRNA reverse: 5′-UUUGUAGGGAUACCAGGCAtt-3′. The above sequence was labeled with green fluorescent FAM.

### Preparation of NP(saMyoD)

NPs and saRNA encapsulated NPs were prepared by double emulsion method with a carrier material PEG-PLA and cationic lipid BHEM-Chol. PEG_5000_-PLA_25000_ and BHEM-Chol are dissolved in 0.5 ml chloroform, 25 μl saRNA solution (including 200 μg saMyoD), and the initial emulsion is formed under the ultrasonic of the probe type ultrasonic breaker (output power 80 W, 30 s). The initial emulsion was added to 1.5 ml of 1% polyvinyl alcohol aqueous solution, and then emulsified again (output power 80 W, 2 min). Then the emulsion was added to 25 ml of 0.3% PVA aqueous solution and the organic solvent was volatilized under reduced pressure, centrifuged (4°C, 30,000g, 1 h). The NPs were collected and suspended twice with ultrapure water, washed by centrifugation, collected. The sample was lyophilized.

### Identification NP Function *In Vitro*

#### Detection of Particles Size, Polydispersity Index, and Zeta Potential

Particles size, polydispersity index and Zeta potential were detected in NP(saMyoD) aqueous solution (1 mg/ml), temperature (25°C) by Nano-ZS90 (Malvern).

#### Measurement of Packing Efficiency and Drug Loading Capacity

1 mg NP(saMyoD) were dissolved in 500 µl TE buffer and 250 µl chloroform. Shaking for 30 min in room temperature. Then centrifuged for 10 min at 13,000 rpm/min, 4°C. At last, NP(saMyoD) were submitted to spectrophotometer to measure the concentration of siRNA. Then, the packing efficiency and drug loading capacity could be calculated.

#### Release *In Vitro*

NP(saMyoD) were dissolved in DEPC water to 1 mg/ml. Then dialysis in PBS (pH7.4) at 100 rpm/min. Collected the dialysate in suitable time and replenish equal volume PBS. Detect the concentration of siRNA with UV method.

### Cell Transfection

ADSCs were seeded in 24-well cell culture plates at a density of 10^5^/well. After overnight culture, the medium was changed to DMEM complete medium (containing 10% FBS) containing FAM-MyoD-saRNA-NPs. The concentration of the particles was 0.366 mg/ml and the FAM-saRNA was 100 nM. The experiments were divided into four groups, ADSCs transfected with PBS group NP), NP(saMyoD) group, NP(scramble) group, and empty NP (Blank) group. The cells were cultured at 37°C for 4 h and then the FAM was detected by Flow cytometry.

### Cellular Viability Assay

ADSCs were incubated with both 100 mg SF(VEGF)/BAMG and 100 mg NP(saMyoD)/BAMG composite scaffold containing DMEM medium, or 200 mg each scaffolds DMEM medium for 3 days (37°C, 5% CO_2)_. The cytotoxicity of 1, 2, and 3 days was detected with an MTT [3-(4,5-dimethylthiazolyl-2)-2,5-diphenyltetrazolium bromide] based *In Vitro* Toxicology kit (Sigma-Aldrich, Schnelldorf, Germany) according to the manufacturer’s instructions.

### Preparation of Silk Fibroin NPs-VEGF

Following the former research (Wang Q. et al., 2017), 200 μl VEGF solution (20 μg/ml) and 8 ml ethanol were added into 20 ml 3% SF aqueous solution. Then, the mixture were stabilized for 5 min under low speed stirring, and frozen at −20°C for 24 h. After ultracentrifugation at 40,000g, the supernatant was removed, and the precipitated silk fibroin particles were lyophilized for further use.

### Scaffold Preparation

The bladder tissue was cut longitudinally from the rat bladder, Then, carefully peeled off the bladder mucosa, and remove the muscle layer of the pulp by microscopy. After soaking in nuclease-free water for 24 h, the treated tissues were dipped in a decellularized solution (0.1% Triton X-100 and 0.15% ammonia water) for 14 days, the decellularized solution was updated every 3 days. Then, the tissues were frozen at −80°C for 24 h, vacuum dried for 24 h, and stored in 75% alcohol.

SF(VEGF) or NP(saMyoD) were dissolved in PBS to a final concentration of 1% and added to BAMG. Due to ultrasound could improve the kinetic energy of biomolecules and increase the collision frequency with BAMG (Zhang et al., 2018), the BAMG was ultrasonically shaken in SF(VEGF) and NP(saMyoD) solution for 10 min and dried under low temperature pressure. Then the SF(VEGF)/BAMG or NP(saMyoD)/BAMG composite scaffold were well prepared. To get the ADSCs well attached the composite scaffold, 100 µl third generation of ADSCs were incubated with SF(VEGF)/BAMG or NP(saMyoD)/BAMG composite scaffold at 3×10^6^/ml. Two hours later serum-free medium was added, 4 h later, the cells were incubated with medium supplemented with serum. After 24 h, the cells were washed with serum medium and washed twice with PBS to remove unadhered and dead cells.

Scaffold mechanical testing: In brief, five pieces BAMGs were picked randomly and immersed in PBS for 24 h. Then those BAMGs were fixed for tensile test with no shift. Before the test, the length and width within the framework were measured. The frame was stretched in the vertical shift in longitudinally with 10mm/min. We reaped these indexes, the initial elastic modulus (EM), ultimate tensile strength (UTS), and % elongation to failure (ETF) with instron 5542 Tensile Tester (Norwood, MA, USA).

### Experimental Animals

Eight-week-old adult male SD rats were acclimatized and housed in cages with free access to food and water in temperature-controlled, pathogen-free animal room facilities (20°C–22°C, humidity 40%–70%, 12 h day/night cycle) for one week before the experiments. The animals were randomly divided into four groups (12 in each group): the sham operation group, the ADSC-BAMG group, the ADSC-saMyo/BAMG group, the ADSC-saMyoD/SF(VEGF)/BAMG group. All animal procedures were approved and supervised by the Ethical Committee of Shanghai Jiaotong University and were performed as the guidelines of the China Act on Welfare and Management of Animals.

### Surgical Procedures for Bladder Augmentation

Rat bladder enlargement model was simply described as follows: rats were anesthetized by intraperitoneal injection of pentobarbital (30 mg/kg), and incised under sterile conditions with a 1-cm incision at the lower abdomen skin to expose the bladder. Cut about 40% to 60% bladder tissue in squares (Wang Q. et al., 2017). BAMG with the same size and area was sutured with the 5–0 polypropylene line to repair the defect of the bladder. BAMG was faced to the bladder cavity. Intravesical incision, normal saline was injected into the bladder from the urethra, and the abdominal cavity was closed after no clear leakage.

### Western Blot Analysis

Total protein extracted from cell were loaded on to sodium dodecyl sulfate‐polyacrylamide gel electrophoresis (SDS‐PAGE) and then transferred onto polyvinylidene fluoride (PVDF) membranes (Millipore, Bedford, MA, USA). The membranes were incubated first with primary antibody and then with secondary antibody. GAPDH was used as a control to verify the equal loading of proteins. Western blot analysis was carried out by a standard protocol using antibodies for SM22a (ab14106, 1:800); Desmin (#5332, 1:1000); α‐SMA (sc-53142, 1:800) and GAPDH (sc-32233, 1:1000)

### Real‐Time RT‐PCR Analysis

Total RNA was extracted from cells using the Trizol reagent (Invitrogen, Thermo Fisher Scientific Inc., NY, USA) as the manufacture’s protocol. The cDNA was acquired by cDNA Reverse Transcription Kit (ABI). Then, the mRNA expression of a-SMA, SM22α and Desmin were conducted by ABI Prism 7900 (Applied Biosystems, Foster City, CA, USA). All reactions were run in triplicate. The cycle threshold (Ct) method was used to calculate values. Target mRNA expression levels were normalized to the housekeeping genes of GAPDH as an internal control.

### VEGF Release *In Vitro*

One hundred milligram of SF(VEGF)/BAMG was suspended in 1 ml of PBS and shakened at 37°C at 100 rpm. A 0.2 ml sample was taken daily from day 1 to day 7 and replenished with an equal volume of fresh PBS. The taken sample concentration of VEGF was measured by ELISA.

### Histopathological and Immunohistochemistry Analysis

Following Rats sacrifice, the bladder tissue was harvested and kept in neutral buffered formalin 10% (v/v) for one day until paraffin embedding for histology. The tissue was sliced in a 5-μm thickness in paraffin block and stained with routine hematoxylin and eosin solutions. Images were captured with a light microscope (Olympus CKX41 microscope, Tokyo, Japan) linked to a computer.

Paraffin-embedded tissue sections were dewaxed with xylene and rehydrated through an ethanol gradient into water. Following blocking of endogenous peroxidase activity with 0.3% hydrogen peroxide for 10 min, the tissue slices were washed with PBS, and incubated with VEGF (sc-80442, 1:200), a-SMA (sc-53142, 1:100) antibody. Next, slices were incubated with biotinylated secondary antibody and then with horseradish peroxidase labeled streptavidin. Diaminobenzidine (DAB) was used as chromogen and antibodies were diluted in the recommended antibody diluting buffer.

### Statistical Analysis

All data are expressed as the mean ± standard deviation. According to different data set, using a two-tailed Student’s t-test or one-way analysis of variance (ANOVA) with the Bonferroni *post hoc* test in GraphPad Prism 8 (GraphPad Software Inc., San Diego, CA, USA). *P*< 0.05 indicates statistical significance.

## Results

### Identification of Rat ADSC

**Figure 1:**
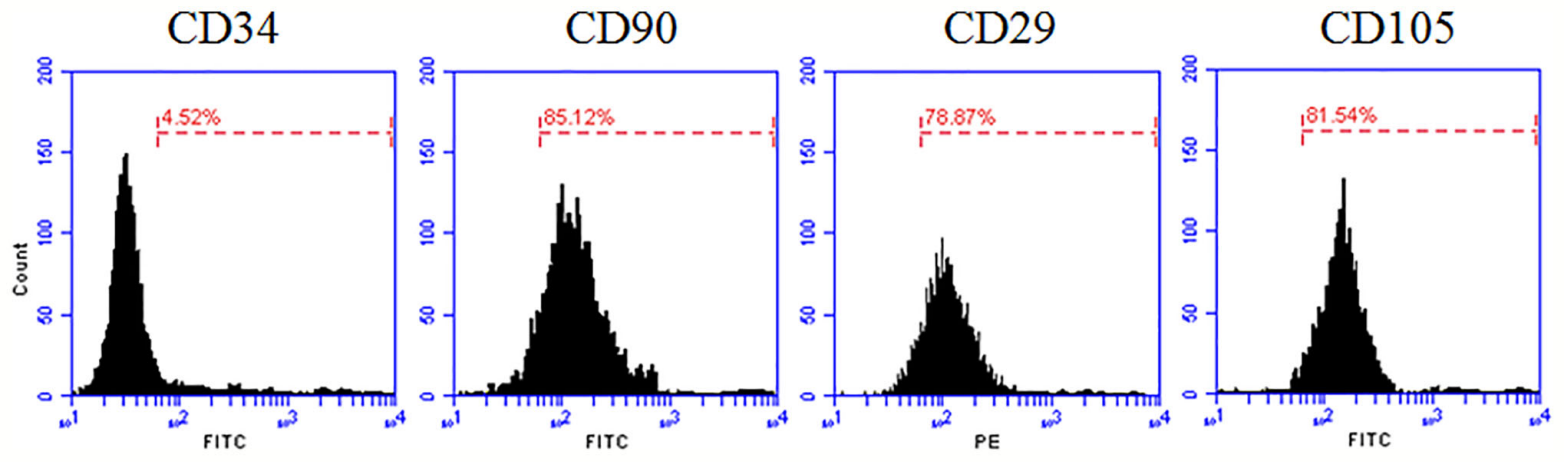
Flow cytometry analysis of rat ADSC. Rat ADSC within two passages were harvested and detected specific cell surface antigens CD34, CD90, CD29, and CD105 by Flow cytometry.

In this study, we isolated and cultured rat ADSC cells, and found uniform spindle-like structure in the second generation. Optimistically, the cell began to proliferate rapidly. To identify the isolated cells were functional ADSCs, we detected cell surface specific markers with flow cytometry analysis (**Figure 1**). The results showed that 85.12% of the detected isolated cells were able to express CD90, 78.87% express CD29 and 81.54% express CD105 (Schäffler and Büchler, 2007; Yamamoto et al., 2007), but few isolated cells express CD34 (4.52%). These results strongly suggested that we had isolated and culture ADSC successfully.

### Characterization of NP(saMyoD)

**Figure 2:**
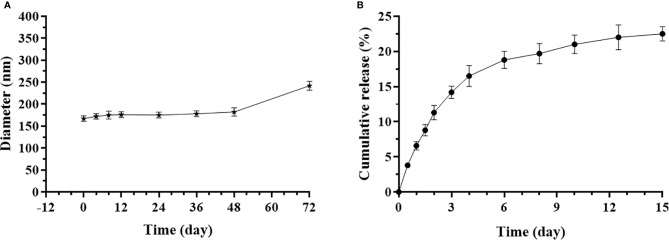
Characterization of NP(saMyoD). **(A)** Detection of the diameter of NP in different time points. **(B)** The cumulative release curve of NP(saMyoD).

The mean diameter of NP(saMyoD) was 167 ± 7.3nm, with PDI 0.208, indicating homogeneous distribution of the particles. The NP(saMyoD) has a 12.1 mV potential voltage, instructing NP(saMyoD) could bind with negative charged cell membrane epitope. NP(saMyoD) displayed a stable diameter of within 180 nm in fetal bovine serum within 48 h, showing good stability (**Figure 2A**). In addition, NP(saMyoD) had encapsulate efficiency (92.3%) and drug loading capacity (0.667%), suggesting that siRNA could be encapsulated efficiently in NP. Besides, the release assay showed that NP(saMyoD) release effect could last for 12 days before plateau (**Figure 2B**).

### NP(saMyoD) Is Able to Increase the Expression of Myod in ADSC Cells

**Figure 3:**
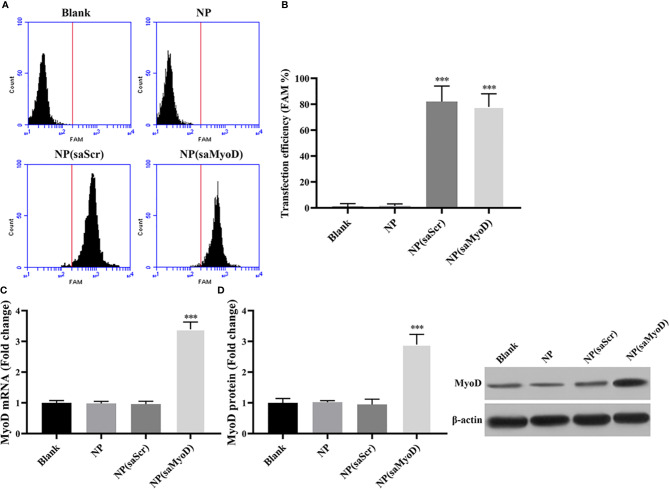
Determination of NP(saMyoD) transfection efficiency in ADSCs. **(A)** Flow cytometry analysis of the NP(saMyoD) transfection efficiency. **(B)** Quantitative analysis of NP(saMyoD) transfection efficiency. **(C)** The mRNA expression level of MyoD. **(D)** The protein expression level of MyoD. ***P < 0.001, compared with the NP(saScr) group.

Myogenic differentiation 1 (MoD) plays a vital role in muscle cell differentiation and muscle regeneration (Weintraub et al., 1991; Clemen et al., 1999; Gaudet et al., 2011). After isolated rat ADSCs *in vitro* efficiently, we were looking for a method to overexpression Myod in ADSCs more efficiently and keep those ADSCs well-functioned in animal body. Currently, rushing research have shed light on the application of NPs in conjunction with small molecular compounds or nucleotide in the medical area (Moghimi et al., 2001; Fan et al., 2018; Li et al., 2019). In this work, we have developed an saMyod-conjugated-hollow gold NPs NP(saMyoD) and identified its biological function. First, FAM-labeled saMyoD was used to prepare NP(saMyoD), we then transfected the compound into ADSCs. The flow cytometry results showed that NP(saMyoD) was greatly expressed in ADSCs, the same to NP(saScr) (**Figure 3A**). The quantitative results showed that about 80% of the cells could be transfected with NP(saMyoD) (**Figure 3B**). Two days after transfection, the expression of MyoD was detected by RT-PCR (**Figure 3C**) and Western Blot (**Figure 3D**). These results demonstrated that, after transfection of ADSC with NP(saMyoD), the mRNA and protein expression of MyoD increased dramatically in ADSCs transfected with NP(saMyoD) compared to other groups (**Figures 3C, D**). Meanwhile, the expression of MyoD in cells transfected with NP was similar to NP (scramble) transfected cells (**Figures 3C, D**).

### Evaluate the Effect of NP(saMyoD) on ADSC

**Figure 4:**
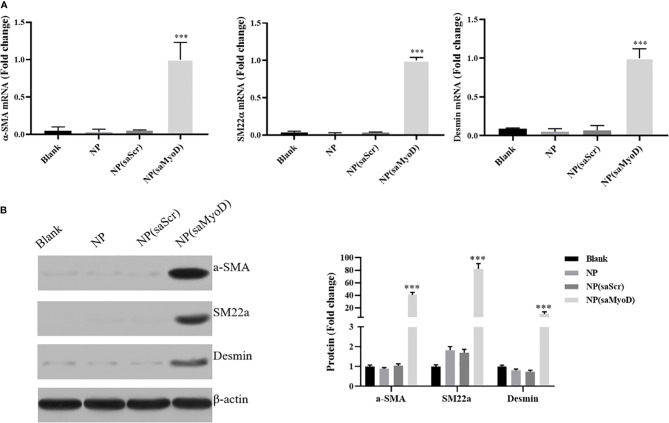
The Expression changes of α-SMA, SM22α, and Desmin were detected. **(A)** The mRNA level of α-SMA, SM22α, and Desmin by RT-PCR. **(B)** The protein level of α-SMA, SM22α, and Desmin by Western blot. ***P < 0.001, compared with the NP(saScr) group.

As the NP(saMyoD) transfected ADSCs showed high Myod expression level, we further investigate the function of those ADSCs. First, we examined the expression level of smooth muscle cell markers α-SMA, SM22α, and Desmin. As shown in **Figure 4A**, compared with NP(saScr), the mRNA expression of α-SMA, SM22α, and Desmin were significantly upregulated after 14 days transfection with NP(saMyoD). The protein expression of α-SMA, SM22α, and Desmin were consistent with mRNA results (**Figure 4B**).

### Evaluation of the ADSC-NP(saMyoD)/SF(VEGF)/BAMG

**Figure 5:**
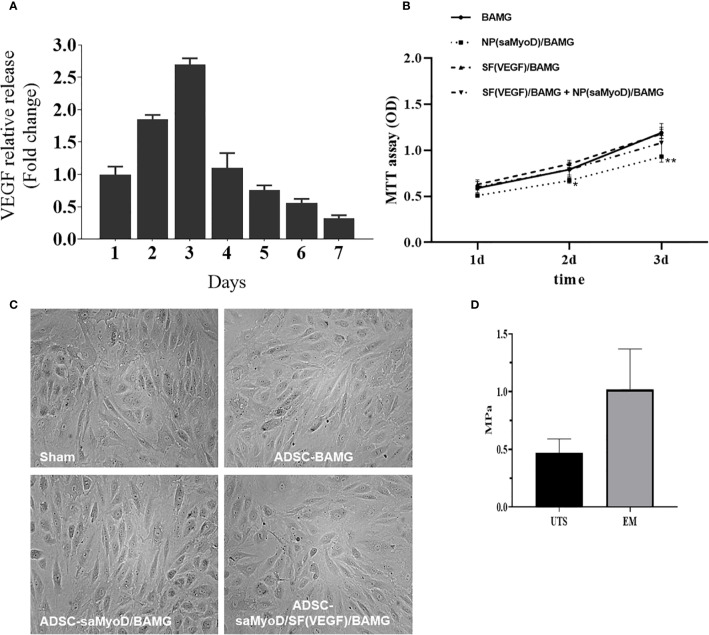
NP(saMyoD)/BAMG and SF(VEGF)/BAMG scaffolds were evaluated. **(A)** The continuous release of VEGF on SF-BAGM. **(B)** The activity of ADSC cells by MTT assay. *P < 0.05, **P < 0.01, compared with the BAGM group. **(C)** The cell morphology of every group. **(D)** The biomechanics of ADSC-NP(saMyoD)/SF(VEGF)/BAMG.

We have evaluated the efficiency and function of NP(saMyoD) on ADSCs, then we’d like to evaluate the SF(VEGF) scaffolds by detecting the sustained release of VEGF on SF-BAGM. The release of VEGF was increased along with the time elapsed and exhibited a burst release in the third day, and then the release ratio of VEGF was slowed down but the release period lasted for more than 7 days (**Figure 5A**). Then, we have checked other indicators in previous reports, the characterization of SF, BAMG and their composite scaffolds were measured with scanning electron microscopy (Zhu et al., 2016). These results indicated that SF-BAMG was successfully constructed and suitable for drug delivery and release regulation. We performed mechanical test for the scaffolds that we generated, the results showed the biomechanics of ADSC-NP(saMyoD)/SF(VEGF)/BAMG is UTS 0.47 ± 0.12 MPa, EM 1.02 ± 0.35 MPa (**Figure 5D**). MTT assay was performed to determine the toxic effects ofNP(saMyoD)/BAMG and SF(VEGF)/BAMG scaffolds on ADSCs. Comparing to the blank group, NP(saMyoD)/BAMG slightly inhibited the proliferation of ADSC, which is consistent with our previous study (Wang et al., 2015), but NP(saMyoD)/BAMG is not toxic accroding to the China national standards for medical devices evaluation (GB/T16886.5-2017), because the inhibition ratio is lower than 20%. The cell morphology is normal under the microscope (**Figure 5C**). SF(VEGF)/BAMG is also not toxic to ADSCs and even promotes the growth of ADSC compared to control group (**Figure 5B**).

### *In Vivo* effect of ADSC-NP(saMyoD)/BAMG and ADSC-SF(VEGF)/BAMG

**Figure 6:**
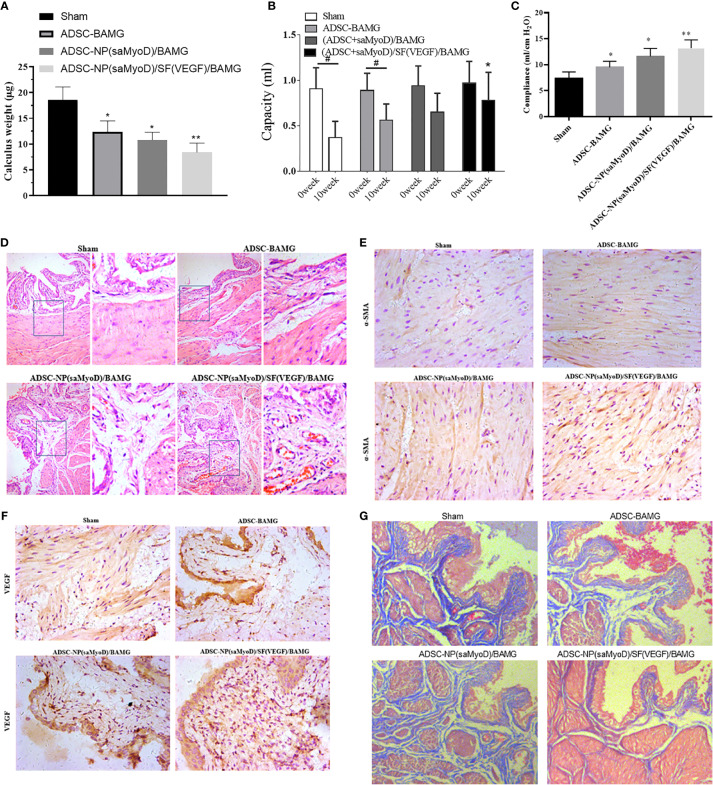
ADSC-NP(saMyoD)/SF(VEGF)/BAMG promotes bladder repair in rat. **(A)** Calculus weight. **(B)** Bladder capacity. **(C)** Bladder compliance. **(D)** HE staining of the bladder regeneration area. **(E)** Immunohistochemistry of α-SMA in bladder tissue. **(F)** Immunohistochemistry of VEGF in bladder tissue. **(G)** Masson dye. *P < 0.05, **P < 0.01, compared with the sham group; ^#^*P* < 0.05, compared with the 0 week group.

A rat model of bladder enlargement was prepared and different scaffolds were implanted. After 10 weeks, the animals were executed to test the repair function of ADSC-NP(saMyoD)/BAMG and ADSC-NP(saMyoD)/SF(VEGF)/BAMG. First, we did not found obvious deterioration in liver and renal of these groups. After detecting the stone situation of these groups, the data demonstrated the composite group showed the least stones than other groups (**Figure 6A**). Then, Bladder function was evaluated, the data showed that the bladder capacities of the ADSC-NP(saMyoD)/SF(VEGF)/BAMG groups were significantly higher than sham group (**Figure 6B**). ADSC-NP(saMyoD)/BAMG also appeared to play a role in bladder repair, but there was no statistical difference compared with sham group (**Figure 6B**). The gradually increasing bladder capacities indicated that MyoD and VEGF might improve the balance between the degradability and generation of the scaffold. The bladder compliance of three treated groups were gradually increased and the compliance of ADSC-NP(saMyoD)/SF(VEGF)/BAMG group was the max (**Figure 6C**).

The HE staining examination of bladder tissue slices in the ADSC-NP(saMyoD)/BAMG and ADSC-NP(saMyoD)/SF(VEGF)/BAMG groups illustrated in-growth of connective tissue into both the marginal and central regions of the original implantation sites. In addition, the entire urothelium regenerated well and more densely and regularly arranged smooth muscle fibers were detected in the ADSC-NP(saMyoD)/SF(VEGF)/BAMG groups (**Figure 6D**). Immunohistochemistry were performed to analyze the regeneration of bladder wall components. The results demonstrated that, comparing with ADSC-NP(saMyoD)/BAMG, there were more α-SMA and VEGF positive signal in ADSC-NP(saMyoD)/SF(VEGF)/BAMG group (**Figures 6E, F**). Thus, ADSC-NP(saMyoD)/SF(VEGF)/BAMG may better promote the growth of smooth muscle and blood vessels formation. At last, the masson dye results showed the sham group showed more collagen than ADSC-NP(saMyoD)/BAMG group, and the ADSC-NP(saMyoD)/SF(VEGF)/BAMG group just had little fibrosis, indicating that the composite scaffolds have enough ability to antifibrotic and with great safety (**Figure 6G**).

## Discussion

Bladder tissue engineering is an important research hotspot of urology. However, the bladder, as a complex organ, has vital storage and excretion function. Owing to the requirement of its good compliance, bladder tissue engineering is therefore a promising but still challenging direction to provide a safe alternative mean to gastrointestinal reconstructive techniques (Lam Van Ba et al., 2015).

In this study, based on the preprepared SF-VEGF-NPs, the appropriate MyoD saRNA molecules were screened to develop NP(saMyoD)/BAMG scaffolds. The rat bladder enlargement model was established to verify the effect of ADSCs inoculation of NP(saMyoD)/SF(VEGF)/BAMG composite scaffold to reconstruct bladder structure and functional regeneration. The results showed that NPs carrying saMyoD and VEGF promoted bladder smooth muscle regeneration and neovascularization, thereby promoting bladder defect repair.

Tissue engineering mainly includes three procedures: seed cells, scaffold materials, and regulatory factors. In this study, BAMG was selected as the scaffold material, ADSC was the seed cell, and saMyoD and VEGF were the regulatory factors. At present, there are three main types of scaffold materials for bladder tissue engineering: acellular matrix, collagen, and polymer synthetic materials. These biomaterials have been shown to have good histocompatibility with bladder epithelial cells and smooth muscle cells. The acellular matrix obtained through removing the cellular components in the original tissue by various decellularization methods is rich in collagen. The acellular matrix is nonimmunogenic, and can be degraded *in vivo*, and is increasingly utilized in tissue engineering research (Sacks and Gloeckner, 1999). BAMG, deriving from the bladder and retaining the original three-dimensional scaffold structure of the bladder, is therefore increasingly used, especially in tissue engineering reconstruction of the bladder (Zhu et al., 2011). In the previous study, we vacuum-dried the traditionally preserved BAMG to make the collagen arrangement more well-aligned and the biomechanical properties improved. We performed mechanical test for the scaffolds that we generated, the results showed the biomechanics of ADSC-NP(saMyoD)/SF(VEGF)/BAMG is UTS0.47 ± 0.12 MPa, EM1.02 ± 0.35 MPa, indicating a familiar former reported parameters (Dahms et al., 1998). It was utilized in the reconstruction of varying degree injury of rabbit bladder defects. These results showed that BAMG was an effective scaffold material for bladder defect reconstruction (Zhu et al., 2011).

Seed cells should have a wide range of properties, such as: easily obtained and cultured, well expanded *in vitro*, and with good compatibility with scaffold materials. Traditional tissue engineering techniques employ their own bladder epithelial cells and smooth muscle cells as seed cells (Yoo et al., 1998). However, many pathological conditions restrict the traditional approach of biopsy to obtain autologous adult cells as a source of seed cells (Dozmorov et al., 2007). Stem cells, with self-renewal activity, high proliferation and multi-directional differentiation potential, are ideal sources of seed cells. In recent years, stem cells, such as bone marrow mesenchymal stem cells, adipose stem cells, endothelial progenitor cells, smooth muscle progenitor cells, have been widely used as seed cells for tissue engineering work. Among them, ADSCs are getting more and more attention (Zhang et al., 2016). Compared with other stem cells, ADSCs, extracting from human body adipose tissue with less trauma and no ethical problems, have higher proliferation rate and differentiation ability than other adult stem cells (Lue et al., 2010; Wang C. et al., 2017; Pokrywczynska et al., 2019). In addition to expressing the myogenic marker MyoD and myosin heavy chain *in vitro* culture (Mizuno et al., 2002), ADSCs have also demonstrated expresses α-actin *in vivo* experiments (Jack et al., 2005), indicating that ADSCs have the potency to form normal smooth muscle. In the previous study, we used adipose stem cells as seed cells combined with BAMG surface for the reconstruction of partial rabbit bladder defects. The results showed that adipose-derived stem cells grew well on BAMG surface, and they have good biocompatibility. In addition, we did not conduct cell tracking for the ADSCs and we could not identify whether the degradation of the scaffolds is in company with the proliferation and differentiation of the ADSCs. But this should get great concern in the further study. And by histological examination, the resulted shown that adipose-derived stem cells can promote the regeneration of the bladder, especially the regeneration of smooth muscle tissue, which is an ideal seed cell of bladder tissue engineering.

The key to tissue engineering reconstruction of the bladder is rapid vascularization and regeneration of bladder smooth muscle. The use of composite growth factors on scaffold materials provides an option for adequate rapid vascularization and regeneration of bladder smooth muscle in tissue engineered bladders. MyoD and VEGF are key factors that promote smooth muscle regeneration and angiogenesis, respectively. This study selected MyoD as a myogenic regulatory factor. However, the current common method is to simply enrich the growth factor on the material, which is very inadequate (Kanematsu et al., 2007). Using genetic engineering technology, seed cells carrying MyoD gene, upregulate the expression of associated growth factors, further promoting the regeneration of bladder smooth muscle by continuous secretion of MyoD growth factor. MyoD can significantly improve the survival rate and functional regeneration of tissues (Lazarous et al., 1996). SaRNA displayed high specificity and low toxicity, making it a new tool for gene function research and clinical treatment (Li et al., 2006; Li, 2008; Wang et al., 2008; Portnoy et al., 2011). In this study, we designed and screened appropriate dsRNA molecules targeting MyoD and transfected them into ADSCs, and found that saMyoD induced high expression of smooth muscle markers α-SMA, SM22a and Desmin in ADSCs.

The effective delivery of the target gene by saRNA is an important part of drug efficacy. The ideal gene inducing vector should be characterized by high efficiency, stability, nontoxicity, convenient preparation and efficient targeting (El-Aneed, 2004; Hughes, 2004). In recent years, nanogene introduction vectors have attracted extensive attention due to their advantages of low toxicity, nonimmunogenicity, large loading capacity, and ease of preparation (Brannon-Peppas and Blanchette, 2004; Yang et al., 2015). Moo Rim Kang (Kang et al., 2012) performed lipid nanoparticle-mediated saRNA treatment in a mouse model of bladder cancer, urinary epithelial absorption, prolonged mouse survival, and tumor regression reached 40%. Lipid nanoparticles are currently the most developed carrier. In this study, lipid nanomaterials were selected as the saMyoD inducing vector. The bonding of saMyoD to the lipid nanomaterials allows it being maintained at the treatment site more permanently, limiting its spread and forming a targeted sustained release system. This method can reduce the potential risk of free diffusion of cytokines to the body, and can make efficient utilization of cytokines.

## Conclusion

In this study, based on the preprepared silk fibroin-VEGF NPs, the saRNA-MyoD lipid NPs were prepared by genetic engineering, and combined with BAMG to form a composite scaffold and inoculated into ADSCs. *In vivo* studies have found that saRNA-MyoD lipid NPs and VEGF silk fibroin NPs promote sustained release of VEGF and MyoD and rapid vascularization and smooth muscle regeneration with scaffold degradation in rats. Through this study, we have further explored new bladder reconstruction materials and factors that facilitate graft smooth muscle regeneration, and desire to provide a new perspective for finding alternative materials for bladder defects.

## Data Availability Statement

All datasets generated for this study are included in the article/supplementary material.

## Ethics Statement

The animal study was reviewed and approved by the Animal Care and Use Committee at Shanghai Jiao Tong University Affiliated Sixth People’s Hospital.

## Author Contributions

These studies were conceived of and designed by all authors. Experiments were performed by CJ, BG, and WZhao with the help of WZ. Data analysis, data interpretation, manuscript preparations were done by CJ, NC, JN, WZhao, BG, and WZhu.

## Funding

This study was supported by the National Natural Science Foundation of China (Grant no. 81200497).

## Conflict of Interest

The authors declare that the research was conducted in the absence of any commercial or financial relationships that could be construed as a potential conflict of interest.

## Abbreviations

ADSC, adipose tissue-derived stem cells; ADSC-BAMG, bladder acellular matrix graft seeded with ADSCs; BAMG, bladder acellular matrix graft; NP, nanospheres; NP(saMyoD)/BAMG, nanosphere encapsulated with small MyoD activating RNA-bladder acellular matrix graft scaffold; NP(saMyoD)/SF(VEGF)/BAMG, NP(saMyoD)/BAMG combine with SF(VEGF); SF(VEGF), encapsulated vascular endothelial growth factor (VEGF) with silk fibroin (SF) fiber.

## References

[B1] Adamowicz J. Kuffel B. Pokrywczynska M. Vontelin Van Breda S. Drewa T. (2019). Reconstructive urology and tissue engineering: converging developmental paths. J. Tissue Eng. Regener. Med. 13 (3). 10.1002/term.2812 30658008

[B2] Ardeshirylajimi A. Ghaderian S. M. Omrani M. D. Moradi S. L. (2018). Biomimetic scaffold containing PVDF nanofibers with sustained TGF-beta release in combination with AT-MSCs for bladder tissue engineering. Gene 676, 195–201. 10.1016/j.gene.2018.07.046 30030200

[B3] Brannon-Peppas L. Blanchette J. O. (2004). Nanoparticle and targeted systems for cancer therapy. Adv. Drug Delivery Rev. 56, 1649–1659. 10.1016/j.addr.2004.02.014 15350294

[B4] Chen W. Shi C. Hou X. Zhang W. Li L. (2014). Bladder acellular matrix conjugated with basic fibroblast growth factor for bladder regeneration. Tissue Eng. Part A 20, 2234–2242. 10.1089/ten.tea.2013.0730 24483233 PMC4137349

[B5] Clemen C. S. Hofmann A. Zamparelli C. Noegel A. A. (1999). Expression and localisation of annexin VII (synexin) isoforms in differentiating myoblasts. J. Muscle Res. Cell Motil. 20, 669–679. 10.1023/A:1005524623337 10672515

[B6] Coutu D. L. Mahfouz W. Loutochin O. Galipeau J. Corcos J. (2014). Tissue engineering of rat bladder using marrow-derived mesenchymal stem cells and bladder acellular matrix. PloS One 9, e111966. 10.1371/journal.pone.0111966 25437001 PMC4249849

[B7] Dahms S. E. Piechota H. J. Dahiya R. Lue T. F. Tanagho E. A. (1998). Composition and biomechanical properties of the bladder acellular matrix graft: comparative analysis in rat, pig and human. Br. J. Urol. 82, 411–419. 10.1046/j.1464-410X.1998.00748.x 9772881

[B8] Dozmorov M. G. Kropp B. P. Hurst R. E. Cheng E. Y. Lin H. K. (2007). Differentially expressed gene networks in cultured smooth muscle cells from normal and neuropathic bladder. J. Smooth Muscle Res. 43, 55–72. 10.1540/jsmr.43.55 17598958

[B9] El-Aneed A. (2004). An overview of current delivery systems in cancer gene therapy. J. Control Release 94, 1–14. 10.1016/j.jconrel.2003.09.013 14684267

[B10] Fan K. Xi J. Fan L. Wang P. Zhu C. Tang Y. (2018). In vivo guiding nitrogen-doped carbon nanozyme for tumor catalytic therapy. Nat. Commun. 9, 1440–1440. 10.1038/s41467-018-03903-8 29650959 PMC5897348

[B11] Gaudet P. Livstone M. S. Lewis S. E. Thomas P. D. (2011). Phylogenetic-based propagation of functional annotations within the Gene Ontology consortium. Brief Bioinform. 12, 449–462. 10.1093/bib/bbr042 21873635 PMC3178059

[B12] Hughes R. M. (2004). Strategies for cancer gene therapy. J. Surg. Oncol. 85, 28–35. 10.1002/jso.20001 14696084

[B13] Jack G. S. Almeida F. G. Zhang R. Alfonso Z. C. Zuk P. A. Rodriguez L. V. (2005). Processed lipoaspirate cells for tissue engineering of the lower urinary tract: implications for the treatment of stress urinary incontinence and bladder reconstruction. J. Urol. 174, 2041–2045. 10.1097/01.ju.0000176489.96993.84 16217390

[B14] Kabadi A. M. Thakore P. I. Vockley C. M. Ousterout D. G. Gibson T. M. Guilak F. (2015). Enhanced MyoD-induced transdifferentiation to a myogenic lineage by fusion to a potent transactivation domain. ACS Synth. Biol. 4, 689–699. 10.1021/sb500322u 25494287 PMC4475448

[B15] Kanematsu A. Yamamoto S. Ogawa O. (2007). Changing concepts of bladder regeneration. Int. J. Urol. 14, 673–678. 10.1111/j.1442-2042.2007.01768.x 17681053

[B16] Kang M. R. Yang G. Place R. F. Charisse K. Epstein-Barash H. Manoharan M. (2012). Intravesical delivery of small activating RNA formulated into lipid nanoparticles inhibits orthotopic bladder tumor growth. Cancer Res. 72, 5069–5079. 10.1158/0008-5472.CAN-12-1871 22869584

[B17] Kocaefe Y. C. Israeli D. Ozguc M. Danos O. Garcia L. (2005). Myogenic program induction in mature fat tissue (with MyoD expression). Exp. Cell Res. 308, 300–308. 10.1016/j.yexcr.2005.03.038 15921681

[B18] Lam Van Ba O. Aharony S. Loutochin O. Corcos J. (2015). Bladder tissue engineering: a literature review. Adv. Drug Delivery Rev. 82-83, 31–37. 10.1016/j.addr.2014.11.013 25446136

[B19] Lazarous D. F. Shou M. Scheinowitz M. Hodge E. Thirumurti V. Kitsiou A. N. (1996). Comparative effects of basic fibroblast growth factor and vascular endothelial growth factor on coronary collateral development and the arterial response to injury. Circulation 94, 1074–1082. 10.1161/01.CIR.94.5.1074 8790049

[B20] Li L. C. Okino S. T. Zhao H. Pookot D. Place R. F. Urakami S. (2006). Small dsRNAs induce transcriptional activation in human cells. Proc. Natl. Acad. Sci. U. S. A 103, 17337–17342. 10.1073/pnas.0607015103 17085592 PMC1859931

[B21] Li W. Yang J. Luo L. Jiang M. Qin B. Yin H. (2019). Targeting photodynamic and photothermal therapy to the endoplasmic reticulum enhances immunogenic cancer cell death. Nat. Commun. 10, 3349–3349. 10.1038/s41467-019-11269-8 31350406 PMC6659660

[B22] Li L. C. (2008). The multifaceted small RNAs. RNA Biol. 5, 61–64. 10.4161/rna.5.2.5989 18398309

[B23] Lue J. Lin G. Ning H. Xiong A. Lin C. S. Glenn J. S. (2010). Transdifferentiation of adipose-derived stem cells into hepatocytes: a new approach. Liver Int. 30, 913–922. 10.1111/j.1478-3231.2010.02231.x 20353420

[B24] Mizuno H. Zuk P. A. Zhu M. Lorenz H. P. Benhaim P. Hedrick M. H. (2002). Myogenic differentiation by human processed lipoaspirate cells. Plast. Reconstr. Surg. 109, 199–209; discussion 210-191. 10.1097/00006534-200201000-00030 11786812

[B25] Moghimi S. M. Hunter A. C. Murray J. C. (2001). Long-circulating and target-specific nanoparticles: theory to practice. Pharmacol. Rev. 53, 283–318. 11356986

[B26] Mousa N. A. Abou-Taleb H. A. Orabi H. (2015). Stem cell applications for pathologies of the urinary bladder. World J. Stem Cells 7, 815–822. 10.4252/wjsc.v7.i5.815 26131312 PMC4478628

[B27] Pokrywczynska M. Jundzill A. Rasmus M. Adamowicz J. Balcerczyk D. Buhl M. (2018). Understanding the role of mesenchymal stem cells in urinary bladder regeneration-a preclinical study on a porcine model. Stem Cell Res. Ther. 9, 328. 10.1186/s13287-018-1070-3 30486856 PMC6260700

[B28] Pokrywczynska M. Rasmus M. Jundzill A. Balcerczyk D. Adamowicz J. Warda K. (2019). Mesenchymal stromal cells modulate the molecular pattern of healing process in tissue-engineered urinary bladder: the microarray data. Stem Cell Res. Ther. 10, 176. 10.1186/s13287-019-1266-1 31196214 PMC6567623

[B29] Portnoy V. Huang V. Place R. F. Li L. C. (2011). Small RNA and transcriptional upregulation. Wiley Interdiscip. Rev. RNA 2, 748–760. 10.1002/wrna.90 21823233 PMC3154074

[B30] Sacks M. S. Gloeckner D. C. (1999). Quantification of the fiber architecture and biaxial mechanical behavior of porcine intestinal submucosa. J. BioMed. Mater. Res. 46, 1–10. 10.1002/(SICI)1097-4636(199907)46:1<1::AID-JBM1>3.0.CO;2-7 10357130

[B31] Schäffler A. Büchler C. (2007). Concise review: adipose tissue-derived stromal cells–basic and clinical implications for novel cell-based therapies. Stem Cells (Dayton Ohio) 25, 818–827. 10.1634/stemcells.2006-0589 17420225

[B32] Vargas K. M. Shon Y. S. (2019). Hybrid lipid-nanoparticle complexes for biomedical applications. J. Mater. Chem. B. 7, 695–708. 10.1039/C8TB03084G 30740226 PMC6363351

[B33] Wang X. Arai S. Song X. Reichart D. Du K. Pascual G. (2008). Induced ncRNAs allosterically modify RNA-binding proteins in cis to inhibit transcription. Nature 454, 126–130. 10.1038/nature06992 18509338 PMC2823488

[B34] Wang C. Chen Z. Wu J. Zhang Y. Hu J. Ge Q. (2015). Small activating RNA induces myogenic differentiation of rat adipose-derived stem cells by upregulating MyoD. Int. Braz. J. Urol. 41, 764–772. 10.1590/S1677-5538.IBJU.2014.0400 26401871 PMC4757007

[B35] Wang C. Liu W. Nie Y. Qaher M. Horton H. E. Yue F. (2017). Loss of MyoD Promotes Fate Transdifferentiation of Myoblasts Into Brown Adipocytes. EBioMedicine 16, 212–223. 10.1016/j.ebiom.2017.01.015 28117277 PMC5474440

[B36] Wang Q. Xiao D. D. Yan H. Zhao Y. Fu S. Zhou J. (2017). The morphological regeneration and functional restoration of bladder defects by a novel scaffold and adipose-derived stem cells in a rat augmentation model. Stem Cell Res. Ther. 8, 149. 10.1186/s13287-017-0597-z 28646909 PMC5482942

[B37] Wang Y. Zhou S. Yang R. Zou Q. Zhang K. Tian Q. (2019). Bioengineered bladder patches constructed from multilayered adipose-derived stem cell sheets for bladder regeneration. Acta Biomater. 85, 131–141. 10.1016/j.actbio.2018.12.016 30553012

[B38] Weintraub H. Davis R. Tapscott S. Thayer M. Krause M. Benezra R. (1991). The myoD gene family: nodal point during specification of the muscle cell lineage. Science 251, 761–766. 10.1126/science.1846704 1846704

[B39] Xiao D. Wang Q. Yan H. Lv X. Zhao Y. Zhou Z. (2017). Adipose-derived stem cells-seeded bladder acellular matrix graft-silk fibroin enhances bladder reconstruction in a rat model. Oncotarget 8, 86471–86487. 10.18632/oncotarget.21211 29156809 PMC5689699

[B40] Yamamoto N. Akamatsu H. Hasegawa S. Yamada T. Nakata S. Ohkuma M. (2007). Isolation of multipotent stem cells from mouse adipose tissue. J. Dermatol. Sci. 48, 43–52. 10.1016/j.jdermsci.2007.05.015 17644316

[B41] Yang X. Iyer A. K. Singh A. Choy E. Hornicek F. J. Amiji M. M. (2015). MDR1 siRNA loaded hyaluronic acid-based CD44 targeted nanoparticle systems circumvent paclitaxel resistance in ovarian cancer. Sci. Rep. 5, 8509. 10.1038/srep08509 25687880 PMC4330541

[B42] Yoo J. J. Meng J. Oberpenning F. Atala A. (1998). Bladder augmentation using allogenic bladder submucosa seeded with cells. Urology 51, 221–225. 10.1016/S0090-4295(97)00644-4 9495701

[B43] Zhang H. Yu N. Zhou Y. Ma H. Wang J. Ma X. (2016). Construction and characterization of osteogenic and vascular endothelial cell sheets from rat adipose-derived mesenchymal stem cells. Tissue Cell 48, 488–495. 10.1016/j.tice.2016.07.004 27514849

[B44] Zhang D. Cao N. Zhou S. Chen Z. Zhang X. Zhu W. (2018). The enhanced angiogenesis effect of VEGF-silk fibroin nanospheres-BAMG scaffold composited with adipose derived stem cells in a rabbit model. RSC Adv. 8, 15158–15165. 10.1039/C7RA11610A PMC9080003 35541334

[B45] Zhu W. D. Xu Y. M. Feng C. Fu Q. Song L. J. (2011). Different bladder defects reconstructed with bladder acellular matrix grafts in a rabbit model. Urol. A 50, 1420–1425. 10.1007/s00120-011-2627-2 21720832

[B46] Zhu W. Chao F. Zhang X. Qiang F. Song L. Rong C. (2016). The Use of Vascular Endothelial Growth Factor with Silk Fibroin Scaffolds and Bladder Acellular Matrix Grafts to Support Bladder Reconstruction in Rabbit Model. J. Tissue Eng. Regen. M. 6, 493–499. 10.1166/jbt.2016.1466

